# Efficient 2,3-Butanediol Production from Cassava Powder by a Crop-Biomass-Utilizer, *Enterobacter cloacae* subsp. *dissolvens* SDM

**DOI:** 10.1371/journal.pone.0040442

**Published:** 2012-07-05

**Authors:** Ailong Wang, Youqiang Xu, Cuiqing Ma, Chao Gao, Lixiang Li, Yu Wang, Fei Tao, Ping Xu

**Affiliations:** 1 State Key Laboratory of Microbial Technology, Shandong University, Jinan, People's Republic of China; 2 School of Life Sciences and Biotechnology, Shanghai Jiao Tong University, Shanghai, People's Republic of China; Centre National de la Recherche Scientifique, France

## Abstract

**Background:**

2,3-Butanediol (BD) is considered as one of the key platform chemicals used in a variety of industrial applications. It is crucial to find an efficient sugar-utilizing strain and feasible carbon source for the economical production of BD.

**Methodology/Principal Findings:**

Efficient BD production by a newly isolated *Enterobacter cloacae* subsp. *dissolvens* SDM was studied using crop-biomass cassava powder as substrate. The culture conditions and fermentation medium for BD production were optimized. Under the optimal conditions, 78.3 g l^−1^ of BD was produced after 24 h in simultaneous saccharification and fermentation (SSF), with a yield of 0.42 g BD g^−1^ cassava powder and a specific productivity of 3.3 g l^−1^ h^−1^. A higher BD concentration (93.9 g l^−1^) was produced after 47 h in fed-batch SSF.

**Conclusions/Significance:**

The results suggest that strain SDM is a good candidate for the BD production, and cassava powder could be used as an alternative substrate for the efficient production of BD.

## Introduction

2,3-Butanediol (BD) is one of the important platform chemicals because of its potential applications in many industrial fields [1]–[3]. For example, as an important starting material, BD can be dehydrated to methyl ethyl ketone (an excellent organic solvent for resins and lacquers), and to 1,3-butadiene for the manufacture of synthetic rubber [3]. Due to the shortage of fossil fuels and the development of biorefinery from renewable resources, microbial production of BD has received great attentions recently [4]–[8]. Technologies for microbial production of BD by *Klebsiella* and *Serratia* strains have been developed in recent years, which promote the progress of biotechnologies for alternative production of oil derivatives [9], [10]. Ma et al [11] optimized the medium for the BD production by a *K. pneumoniae* strain, and a high BD concentration of 150 g l^−1^ with a diol productivity of 4.2 g l^−1^ h^−1^ was reached using glucose as substrate. Zhang et al [12] developed a mutant of *S. marcescens*, and the high BD concentration (152 g l^−1^) and productivity (2.7 g l^−1^ h^−1^) were reached from sucrose. Moreover, Ji et al [13] constructed a *K. oxytoca* mutant, and the BD concentration of 130 g l^−1^ and productivity of 1.6 g l^−1^ h^−1^ were reached from glucose. However, these studies used the refined glucose and sucrose as substrates, which may not be so economically reasonable [4], [11], [12], [14]. To minimize the production cost of BD, low price substrates and efficient strains were still required [10]. Therefore, the renewable bioresources, such as the lignocellulosic materials, corncob molasses and corncob acid hydrolysate [8], [15], Jerusalem artichoke tubers hydrolysate [16] have attracted attentions for the BD production from an economic point of view.

Cassava (*Manihot esculenta*) is a relatively inexpensive and available starch crop-biomass widely planted in tropical and subtropical areas. It is one of the most efficient crops in term of carbohydrate production, and the roots of cassava are rich in starch. Cassava starch, cassava powder, cassava bagasse, cassava chips, and fresh cassava roots have been reported for microbial production of useful chemicals such as ethanol [17]–[19], lactic acid [20], [21], and acetone–butanol [22], [23]. However, little attention has been paid on microbial production of BD from cassava. Therefore, it is of vital necessity to screen promising strains with industrial potential for the biotransformation of cassava to BD.

In the present study, a new strain identified and named *Enterobacter cloacae* subsp. *dissolvens* SDM was isolated for the efficient production of BD using cassava powder as substrate. The cultivation conditions and fermentation medium for the BD production were optimized. Simultaneous saccharification and fermentation (SSF) and fed-batch SSF were performed to obtain high BD production.

## Results

### Enzymatic hydrolysis of cassava powder

Starch materials generally need the pretreatment when they are used as substrates for microbial fermentation. The saccharification conditions of liquefied cassava powder were optimized in 200-ml Erlenmeyer flasks each containing 50 ml of the reaction suspension firstly. The glucoamylase dosage was added from 100 U g^−1^ to 1,000 U g^−1^ cassava powder. At a dosage of 400 U glucoamylase g^−1^ cassava powder, glucose was released completely within 4 h. This glucoamylase usage was selected for further experiments.

### Strain isolation and identification

Strain isolation was carried out using the cassava powder hydrolysate as carbon source. The strain that produced the maximum BD was isolated, and identified by Deutsche Sammlung von Mikroorganismen und Zellkulturen GmbH (DSMZ) in Germany. It was a motile, facultatively-anaerobic, gram-negative and rod-shaped bacterium. Voges–Proskauer test was positive, as well as β-galactosidase, catalase, urease, arginine dihydrolase, and ornithine decarboxylase. However, methyl red test was negative, and the result was same for oxidase reaction, gelatin degradation, and indole production. It was capable of growing on glucose, fructose, mannose, maltose, D-xylose, sucrose, trehalose, L-arabinose, rhamnose, and galactose, and could utilize citrate and malonate. Fatty acid composition analysis was typical for *Enterobacteriaceae*. Sequence analysis of the 16S rDNA gene revealed 100% similarity to *Enterobacter cloacae* subsp. *dissolvens* in the diagnostic regions (GenBank accession number HQ434623). Based on the conventional markers (morphology), chemotaxonomic markers (fatty acid pattern), and molecular biological results (comparative 16S rDNA gene sequencing), it was concluded that this strain belongs to species *E. cloacae* subsp. *dissolvens*, and designated as *E. cloacae* subsp. *dissolvens* SDM. Strain SDM was deposited in DSMZ (DSM 25274) and China General Microbiological Culture Collection (CGMCC 4230). Genome of the strain was also sequenced and released recently [24].

### Effects of temperature and pH of medium on BD production

The efficiencies of bioprocesses are strictly temperature-dependent owing to the strong dependence of enzymatic activity and cellular maintenance upon temperature [25]. In this study, the effects of temperature on cell growth and BD production were examined (Figure S1).

The results showed that 30°C was the most favourable temperature for the BD production, cell growth and glucose utilization. With suboptimal temperature, the rate of metabolism decreased. In a previous research, the optimum temperature for BD production by *E. cloacae* was also found to be 30°C [26].

The effect of the initial pH (5.0−8.5) of medium on BD production was also investigated at 30°C (Figure S2). Gradual increase of cell growth was observed at pH range from 5.0 to 8.5. BD production increased with pH from 5.0 to 7.0, and then BD concentration changed slightly. Consequently, a temperature of 30°C and initial pH of 7.0 were chosen for subsequent fermentations.

### Effects of nitrogen source on cell density and BD production

The effects of nitrogen source on *E. cloacae* subsp. *dissolvens* SDM growth and BD production were studied. Table 1 shows the effects of the low cost nitrogen source on BD production and cell grown. The best results were obtained when corn steep liquor powder (CSLP) was used as nitrogen source. Thus CSLP was selected as the optimal nitrogen source for further studies. Various CSLP concentrations ranging from 5 to 15 g l^−1^ were used to test the effect of its concentration on the BD production. When the CSLP concentration was 5 to 10 g l^−1^, the BD concentration increased to the maximum value. Therefore, the CSLP concentration of 10 g l^−1^ was selected for further statistical optimization experiments.

**Table 1:** Effects of nitrogen source on BD production and cell growth.

Nitrogen source	BD (g l^−1^)	OD_620_
CSLP	19.9±0.3	26.7±0.5
NH_4_Cl	17.2±0.6	22.2±0.4
Urea	17.8±0.2	22.9±0.4
(NH_4_)_2_SO_4_	15.1±0.4	18.4±0.3
(NH_4_)_2_HPO_4_	18.7±0.3	24.8±0.4
NH_4_NO_3_	17.5±0.5	24.1±0.2

The nitrogen source concentration was 5 g l^−1^. Data are the means ± SDs from three parallel experiments. CSLP: corn steep liquor powder.

### Effect of cassava powder concentration on BD production in SSF

**Figure 1:**
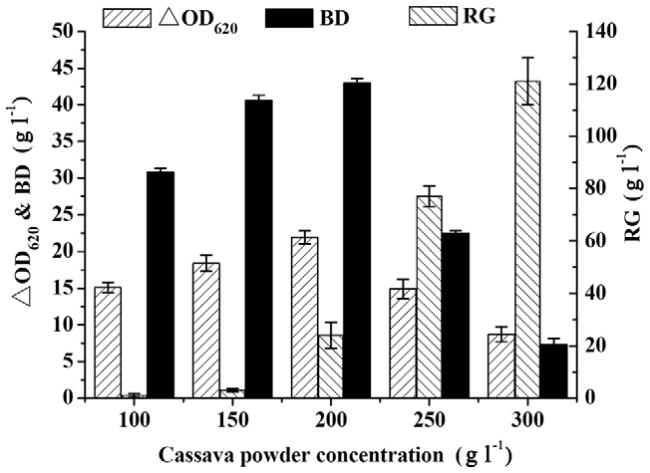
Effect of cassava powder concentration on BD production in SSF. RG: residual glucose; ΔOD_620_: the cell growth, which was determined by the difference value between the measured optical density in the fermentation process and the initial value at the beginning of the fermentation. Data are the means ± SDs from three parallel experiments.

To study the effect of the initial cassava powder concentration on BD production, various concentrations of cassava powder were utilized by strain SDM in SSF process to produce BD. As shown in Figure 1, cell density increased with the cassava powder concentrations to 200 g l^−1^, and then decreased sharply. The production of BD increased significantly with the increase of cassava powder concentrations from 100 to 200 g l^−1^. When the cassava powder concentration was over 200 g l^−1^, the residual sugar increased noticeably and cell density and BD concentration decreased sharply. This result showed that the high initial substrate concentration would affect the metabolism of strain SDM. The reason may be that the high glucose concentration could inhibit the BD production and sugar utilization. The inhibition of high sugar concentration on the cell growth and BD production probably results from a fall in water activity that affects the metabolic rates [27].

### Optimization by central composite design

To obtain the optimal levels of cassava powder, glucoamylase and CSLP for BD production, and investigate the interactions between the three variables, a response surface methodology using central composite design was sequentially employed. Table 2 lists the coded and real values of the variables at various levels. Experimental design and results were presented in Table 3. By applying multiple regression analysis on the experimental data, the second-order polynomial equation is given as Equation 1.
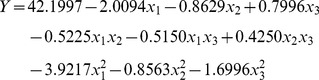
(1)


**Table 2:** Coded and real values of variables in central composition design.

Variable	Levels of variables				
	−1.682	−1	0	1	1.682
*X*_1_: cassava powder (g l^−1^)	115.9	150	200	250	284.1
*X*_2_: glucoamylase (U/g cassava powder)	231.8	300	400	500	568.2
*X*_3_: CSLP (g l^−1^)	6.6	8	10	12	13.4

**Table 3:** Experimental design and results of central composite design.

Run	Factor variables Coded levels			Experimental and predicted results of BD production (g l^−1^)	
	*x*_1_	*x*_2_	*x*_3_	Experimental	Predicted
1	−1	−1	−1	37.05	37.18
2	+1	−1	−1	35.28	35.24
3	−1	+1	−1	36.44	35.65
4	+1	+1	−1	30.77	31.62
5	−1	−1	+1	40.23	38.96
6	+1	−1	+1	34.59	34.96
7	−1	+1	+1	39.51	39.13
8	+1	+1	+1	33.59	33.04
9	−1.682	0	0	33.32	34.49
10	+1.682	0	0	28.30	27.73
11	0	−1.682	0	40.95	41.23
12	0	+1.682	0	38.01	38.33
13	0	0	−1.682	36.34	36.05
14	0	0	+1.682	37.85	38.74
15	0	0	0	41.46	42.20
16	0	0	0	43.54	42.20
17	0	0	0	41.37	42.20
18	0	0	0	42.13	42.20
19	0	0	0	43.32	42.20
20	0	0	0	41.48	42.20

The results of the second-order response surface model in the form of the analysis of variance (ANOVA) are shown in Table 4. The fitness of the model was examined by the coefficient of determination *R^2^*. In this study, *R^2^* was calculated to be 0.9673, indicating that the sample variation of more than 96.7% was attributed to the given independent variables. The value of the adjusted determination coefficient (Adj *R^2^* = 93.78%) was also high to confirm the significance of the model.

**Table 4:** Analysis of variance (ANOVA) for the quadratic model.

Source	Sum of squares	Degree of freedom	Mean of square	*F* value	*P* value
Model	328.1976	9	36.4664	32.8442	<0.0001
*x*_1_	55.1439	1	55.1439	49.6665	<0.0001
*x*_2_	10.1688	1	10.1688	9.1587	0.0128
*x*_3_	8.7309	1	8.7309	7.8636	0.0187
*x*_1_ *x*_2_	2.1841	1	2.1841	1.9671	0.1910
*x*_1_ *x*_3_	2.1218	1	2.1218	1.9110	0.1969
*x*_2_ *x*_3_	1.4450	1	1.4450	1.3015	0.2805
*x*_1_^2^	221.6356	1	221.6356	199.6204	<0.0001
*x*_2_^2^	10.5680	1	10.5680	9.5183	0.0115
*x*_3_^2^	41.6273	1	41.6273	37.4924	0.0001
Residual	11.1029	10	1.1103		
Lack of fit	6.2947	5	1.2589	1.3092	0.3874
Pure error	4.8081	5	0.9616		
Total	339.3005	19			

*R^2^* = 96.73%; Adj *R^2^* = 93.78%.

*P* value less than 0.05 indicates that the model terms are significant.

*P* value less than 0.01 indicates that the model terms are highly significant.

Furthermore, ANOVA for the response surface quadratic model presented in Table 4 also showed that this regression was statistically significant (*P*<0.0001). There was only a 0.01% chance that it could occur due to noise. In addition, the analysis of variance showed that there was a non-significant lack of fit (*P* = 0.3874). The model was found to be adequate for prediction within the range of variables selected.

**Figure 2:**
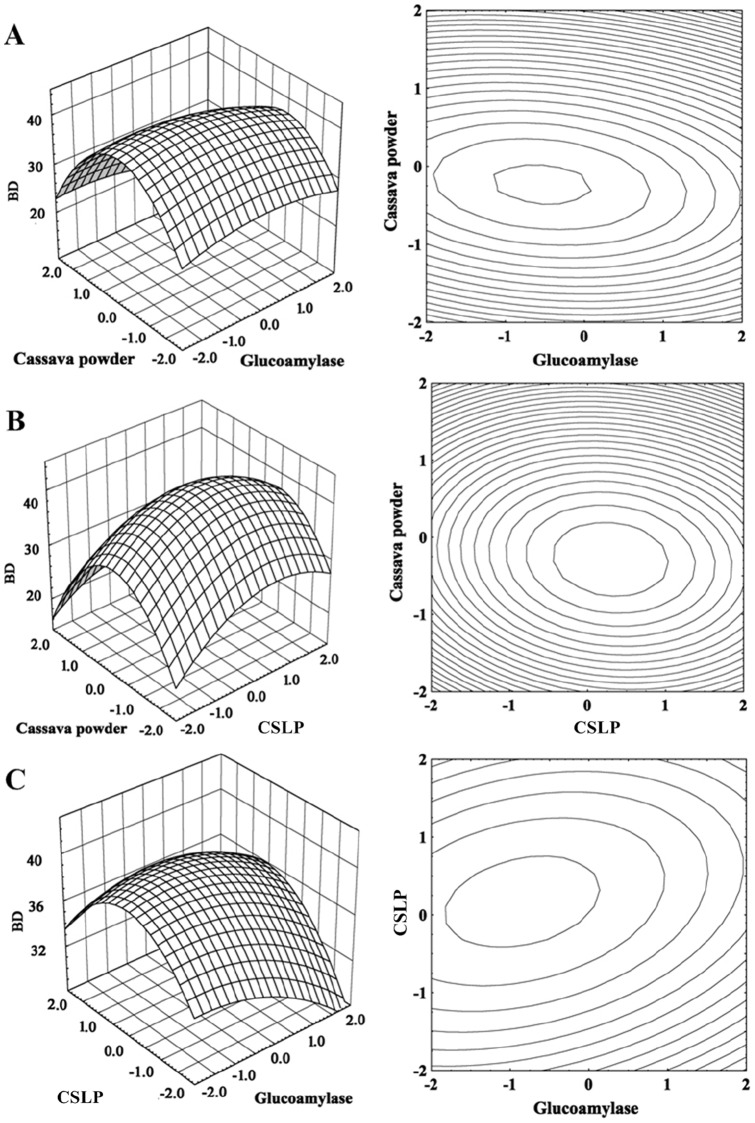
Contour plot fitted to the quadratic model. Effects of cassava powder and glucoamylase (A); effects of cassava powder and CSLP (B); effects of CSLP and glucoamylase (C) on BD production.

The 2D contour plots and the 3D response surface curve by the predicted model were drawn to gain a better understanding of the effects of the variables on BD production. An elliptical contour of the contour plots indicated that the interactions between the independent variables were significant, such as cassava powder and glucoamylase, as well as glucoamylase and CSLP (Figure 2A, C). Figure 2B shows a nearly circular contour, indicating little interaction between cassava powder and CSLP.

The predicted maximum value was identified by the surface confined in the smallest ellipse in the contour diagram. The predicted optimum levels of cassava powder, glucoamylase and CSLP were obtained by applying the regression analysis using SAS. The optimal values of the test variables in coded unit area were *x*_1_ = −0.24621, *x*_2_ = −0.37265, *x*_3_ = 0.22594.

The optimized values were cassava powder, 187.7 g l^−1^; glucoamylase, 363 U g^−1^ cassava powder; CSLP 10.5 g l^−1^. The optimized values were used for further investigation of BD production in SSF process.

### BD production in SSF and fed-batch SSF

**Figure 3:**
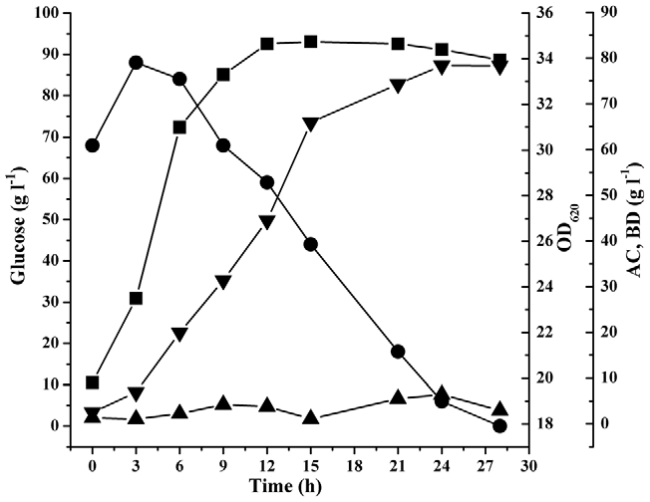
Time course of BD production during SSF in a 5–l stirred bioreactor. ▪: cell density, OD_620_; •: glucose concentration; ▴: AC concentration; ▾: BD concentration. The cultivation was carried out at an initial pH of 7.0. When pH decreased to 6.0, it was maintained at 6.0 in the fermentation processes by automatic addition of 4 M H_3_PO_4_ or 6 M KOH using a program-controlled peristaltic pump. The agitation speed was 500 rpm and aeration rate was 1.0 vvm.

The validation experiment was carried out with SSF under the optimized conditions in a 5–l stirred bioreactor. The maximum BD production was 78.3 g l^−1^ in 24 h with a rate of 3.3 g l^−1^ h^−1^ and a yield of 0.42 g BD g^−1^ cassava powder (Figure 3).

**Figure 4:**
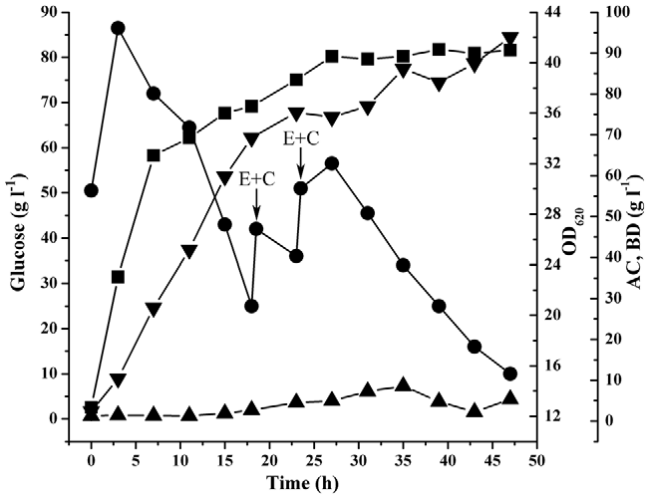
Time course of BD production during fed-batch SSF in a 5–l stirred bioreactor. ▪: cell density, OD_620_; •: glucose concentration; ▴: AC concentration; ▾: BD concentration. E: glucoamylase; C: the clarified solution of cassava powder. The glucoamylase and liquefied solution of cassava powder were added into the bioreactor at appropriate time. The cultivation was carried out at an initial pH of 7.0. When pH decreased to 6.0, it was maintained at 6.0 in the fermentation processes by automatic addition of 4 M H_3_PO_4_ or 6 M KOH using a program-controlled peristaltic pump.

In order to achieve a higher BD production, fed-batch SSF was conducted in the 5–l stirred bioreactor. Fed-batch SSF was started with the optimized conditions (Figure 4). When the sugar concentration was reduced to about 30 g l^−1^, glucoamylase and liquefied solution of cassava powder (500 g l^−1^) were added into the bioreactor. As shown in Figure 4, the BD and acetoin (AC) concentrations were 93.9 g l^−1^ and 5.3 g l^−1^ after 47 h in fed-batch SSF. The ratio of the three stereoisomers of BD produced by *E. cloacae* subsp. *dissolvens* SDM was analyzed by GC, which were 83.1% of *meso*-BD, 5.2% of (2*R*,3*R*)-BD, and 11.7% of (2*S*,3*S*)-BD, respectively (Figure S3).

## Discussion

Most published studies on BD production were using *Klebsiella*, and a few studies were reported using *Enterobacter* [1], [2], [10]. Saha and Bothast [26] previously reported that an *E. cloacae* strain produced 12.0 g l^−1^ of BD in 24 h (0.34 g g^−1^ theoretical sugars) from dilute acid pretreated corn fiber. In the present study, a new *E. cloacae* subsp. *dissolvens* strain SDM was isolated and high production of BD was obtained from cassava powder by fermentation. Besides the cassava, the utilization of glucose, xylose and arabinose, the common sugars that widely exist in lignocellulosic materials, was also investigated using strain SDM in 500-ml flasks. The results showed that all these sugars could be utilized for cell growth and BD production (Table 5). Moreover, a fed-batch fermentation for BD production using glucose was also carried out by strain SDM in 5–l bioreactor (data not shown in details), and high concentration (more than 130 g l^−1^) and yield (95%) of BD were obtained, which were similar to the results of the previous work using a *K. pneumoniae* strain [11]. These productive characteristics demonstrated that strain SDM has a broad substrate spectrum and could ferment the primary pentose and hexose that exist in lignocellulosic materials for the BD production; and it could also produce high concentration of BD from glucose. The recently released genome sequence of strain SDM contains the complete pentose metabolic pathway, which is important for pentose utilization in the industrial application. Besides, there are annotated full genes for other carbohydrates utilization, indicating that strain SDM may have a wide substrate spectrum. The genome sequence also contains a series of membrane transport systems, involved in ATP-binding cassette (ABC) transporters. The ABC transporters play important roles in not only accumulating compatible solutes and substrates but also excreting unwanted products. It may be an important reason for the high-yield production of BD [24]. These qualities of the strain enable it to be used for large-scale BD production like other high-productive strains [11]–[13].

**Table 5:** Utilization of glucose, xylose and arabinose by strain SDM.

Substrate	Concentration (g l^−1^)	OD_620_	BD (g l^−1^)
Glucose	59.8±0.3	24.9±0.5	22.3±0.4
Xylose	60.3±0.2	24.2±0.4	21.4±0.3
Arabinose	60.7±0.5	23.5±0.4	22.7±0.4

Experiments were carried out in 500-ml flasks. The flasks with 100 ml of the fermentation medium were incubated at 30°C on a reciprocal shaker at 100 rpm for 12 h. Data are the means ± SDs from three parallel experiments.

The theoretical yield of BD from glucose is 0.5 g g^−1^, and Qin et al [14] reported the diol yield reached 98% of its theoretical value. In this study, the yield of 0.42 g BD g^−1^ cassava powder corresponds to a theoretical yield of 94% from glucose on the calculation of 1.0 g starch producing 1.1 g glucose [19]. Cassava has content of toxic chemical cyanogen, which may inhibit the metabolism of bacteria [21], [28], [29]. This may be a reason that the yield of BD from cassava powder is lower than that of the BD from glucose.

In the case of a bulk chemical production, the cost of the product is mostly affected by the prices of raw materials. Therefore, low-cost raw materials are essential for the economical production of BD. BD production involves different processes, such as raw material processing for substrates, fermentation, and product separation. Among those, the cost of raw materials accounted for more than about 30% of total production-cost (personal communication, Apple Flavor & Fragrance Group Co. Ltd, Shanghai, China). The unit price of raw cassava powder is more inexpensive than that of the fresh corn and processed cassava products ([21], [30] http://www.fao.org/). The price of cassava powder was estimated about 440 $/t, which was lower than pure glucose (700 $/t) and corn starch (500 $/t). Although the sugars from lignocellulose sources are desirable for the production of biochemicals, the productivity is still low at the current state, which may increase the economical costs [2]. Moreover, the cost of enzymes used in this study for processing a ton of cassava powder was estimated only about 5% of that of cassava powder. This study implies that cassava powder could be used as an alternative economic substrate for BD production.

In summary, in the present study, BD production from cassava powder was investigated by a newly isolated *E. cloacae* subsp. *dissolvens* SDM. Using SSF, 78.3 g l^−1^ of BD was obtained under the optimized conditions, and a higher BD concentration (93.9 g l^−1^) was produced in fed-batch SSF. This study demonstrates that strain SDM is a good candidate for the BD production, and cassava powder could be used as a suitable substrate for the economical production of BD.

## Materials and Methods

### Materials

(2*S*,3*S*)-BD (99.0%), (2*R*,3*R*)-BD (98.0%), and *meso*-BD (98.0%) were purchased from ACROS. Cassava powder used in this study was purchased from Guangxi province (China) by grinding dry cassava chips, and this crude powder was used directly as a raw material for the fermentation process. The starch content of the cassava powder was 80.7% (w w^−1^), with moisture, crude fiber and total proteins content of 10.0% (w w^−1^), 2.9% (w w^−1^) and 3.7% (w w^−1^), respectively. CSLP was purchased from Shanghai Xiwang Starch Sugar CO. Ltd (Shanghai, China), which contained 48.0% (w w^−1^) of proteins, 1.6% (w w^−1^) of glucose, and 6.8% (w w^−1^) of lactic acid. The weight percentages above were all in wet basis. The 0.22 μm filtration membrane used in aqueous phase was purchased from Tianjin Jinteng Experiment Equipment CO. Ltd (Tianjin, China). All other chemicals were of analytical grade available commercially.

### Isolation and strain identification

Farmland soil sample (1 g) was inoculated into 50 ml of an isolation medium (no specific permits were required for the described field studies; the location is not privately-owned or protected in any way; the field studies did not involve endangered or protected species), and incubated at 37°C for 24 h. The isolation medium consisted of cassava powder hydrolysate (glucose concentration in the medium is 50 g l^−1^), peptone 5 g l^−1^, yeast extract 5 g l^−1^, mixed solution A (sodium acetate, 4 g l^−1^; KCl, 0.5 g l^−1^; MgSO_4_·7H_2_O, 0.15 g l^−1^; FeSO_4_·7H_2_O, 0.05 g l^−1^; MnSO_4_·7H_2_O, 0.03 g l^−1^), pH 7.0. One milliliter of the resulting culture was transferred into the same isolation medium and enriched for 24 h under the same conditions. After enrichment, the culture was spread onto the isolation medium plate, and incubated at 37°C for 12 h to get the single colonies.

The single colonies, of which Voges–Proskauer test was positive, were transferred to the isolation medium and incubated at 37°C for 12 h. The culture was then centrifuged at 12,000 × *g* for 10 min under 4°C and the supernatant was used to detect BD concentration. The most dominant strain for BD production was deposited and identified.

### Enzymes and enzymatic hydrolysis

Commercially available α-amylase (40,000 U ml^−1^, produced by *Bacillus licheniformis*) and glucoamylase (100,000 U ml^−1^, produced by *Aspergillus niger*), by Anke Bioengineering Co. Ltd. Shandong Province, were used for the enzymatic hydrolysis of cassava powder.

Enzymatic hydrolysis was performed in two steps, namely, liquefaction and saccharification. The liquefaction was carried out by adding excess α-amylase to the suspension which was adjusted to pH 6.0 using 2 mol l^−1^ of HCl and incubated at 95°C for 2 h in a water bath (100 rpm). After the liquefying steps were completed, the liquefied cassava mash was saccharified by adding appropriate dosage of glucoamylase at pH 4.5 and maintained at 60°C in a water bath (100 rpm), and the resulting saccharified cassava solution was used as the initial carbon source in the fermentation process. For SSF and fed-batch SSF, the liquefied solution was autoclaved and the glucoamylase filtrated by the 0.22 μm filtration membrane was added to the medium with the inoculum for fermentation.

### Culture media and conditions

The strain was maintained on agar slants each containing the following medium: glucose 15 g l^−1^, peptone 10 g l^−1^, yeast extract 5 g l^−1^, KCl 5 g l^−1^, and agar 18 g l^−1^ at pH 7.0. The slants were incubated at 30°C for 12 h and then stored at 4°C.

The seed culture was prepared by inoculating a full loop of cells from freshly prepared slants into 100 ml of the isolation medium. The cultivation was conducted in 500-ml shake flasks for 12 h with agitation (100 rpm, reciprocal shaker) at 30°C. The fermentation was initiated by inoculation with a seed culture (10%, v v^−1^).

The isolation medium was used to study the effects of temperature and initial pH of medium on cell growth and BD production. To investigate the effects of nitrogen sources on BD production, the cassava powder hydrolysate (glucose concentration in the medium is 60 g l^−1^) was added into mixed solution A, and supplemented with nitrogen sources to the desired concentration. To study the effect of cassava powder concentration on BD production in batch SSF, medium containing CSLP (10 g l^−1^), mixed solution A, and 100–300 g l^−1^ of liquefied cassava powder supplemented appropriate dosage of glucoamylase was used. Fermentations were carried out in 500-ml flasks with 100 ml of medium at 30°C.

### Statistical experiment design and data analysis

Response surface methodology was used to optimize the variables and investigate the interactions between the variables based on central composite design with five coded levels. For the statistical calculation, the relationship between the coded values and actual values is described by Equation 2.
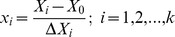
(2)


Where *X_i_* is the real value of the independent variable, *x_i_* is the coded value of the independent variable *X_i_*, *X_0_* is the real value of the independent variable *X_i_* at the center point and Δ*X_i_* is the step change. The second-order model used to fit the response to the independent variables is shown in Equation 3.
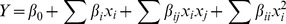
(3)


Where *Y* is predicted response, *β*_0_ is offset term, *β_i_* is linear effect, *β_ii_* is squared effect, *β_ij_* is interaction effect, and *x_i_* is dimensionless coded value of *X_i_*.

The flasks with 100 ml of the fermentation medium were incubated at 30°C on a reciprocal shaker at 100 rpm. All experimental designs were performed at least in triplicate. Statistical and numerical analyses were carried out by means of ANOVA. The quality of the polynomial model equation was judged statistically by the coefficient of determination *R* ^2^. The statistical significance of the model was determined by the Fischer's test. The data was analyzed statistically by using SAS package. Contour presentations were plotted using Statistica software (StatSoft, Tulsa, OK).

### SSF and fed-batch SSF in 5-l bioreactor

In SSF, the glucoamylase filtrated by 0.22 μm filtration membrane was added in portions, and the liquefied solution of cassava powder was added in order to keep an appropriate glucose concentration. The cassava powder concentration added was 188 g l^−1^. The fermentation was initiated by inoculation with a seed culture (10%, v v^−1^), and carried out at 30°C and initial pH of 7.0. When pH decreased to 6.0, it was maintained at this level by automatic addition of 4 M H_3_PO_4_ or 6 M KOH using a program-controlled peristaltic pump.

In fed-batch SSF, the experiments were carried out the same as SSF. Liquefied cassava powder (500 g l^−1^) was added when residual glucose concentration was reduced to about 30 g l^−1^.

Samples of SSF and fed-batch SSF were collected periodically to determine the concentrations of cell density, glucose, AC and BD.

### Analytical methods

The optical density (OD) was measured at 620 nm using a 7200 visible spectrophotometer after an appropriate dilution. The initial optical density of the solution at various concentrations of cassava powder was remarkably different; therefore, the cell growth (ΔOD_620_) was determined by the difference value between the measured optical density in the fermentation process and the initial value at the beginning of the fermentation. Concentrations of glucose, acetate, lactate, succinate and ethanol were determined by HPLC [8]. After extraction with ethyl acetate, BD was quantified by a GC system [8]. The ratio of the three stereoisomers of BD was analyzed by GC [31].

## Supporting Information

Figure S1 **Effects of temperature on cell growth and BD production.** ΔOD_620_: the cell growth, which was determined by the difference value between the measured optical density in the fermentation process and the initial value at the beginning of the fermentation. RG: residual glucose. BD: 2,3-butanediol concentration. Data are the means ± SDs from three parallel experiments. Fermentations were carried out in 500-ml flasks with 100 ml of medium.
(PDF)

Figure S2 **Effects of the initial pH of the medium on cell growth and BD production.** Data are the means ± SDs from three parallel experiments. The medium consisted of cassava powder hydrolysate (glucose concentration in the medium is 50 g l^−1^), peptone 5 g l^−1^, yeast extract 5 g l^−1^, sodium acetate 4 g l^−1^, KCl 0.5 g l^−1^, MgSO_4_·7H_2_O 0.15 g l^−1^, FeSO_4_·7H_2_O 0.05 g l^−1^, MnSO_4_·7H_2_O 0.03 g l^−1^. Fermentations were carried out in 500-ml flasks with 100 ml of medium at 30°C.
(PDF)

Figure S3 **GC analysis of fermented products by** ***E. cloacae*** **subsp.** ***dissolvens*** **SDM.** IA: isoamyl alcohol was used as the internal standard. The ratio of the three stereoisomers of BD was analyzed by GC (Agilent GC6820) using a fused silica capillary column (Supelco Beta DEX^TM^ 120, inside diameter, 0.25 mm; length, 30 m). The operating conditions were as follows: nitrogen was used as the carrier gas; the injector temperature and detector temperature were both 280°C; the column oven was maintained at 40°C for 3 min and then programmed to increase to 80°C at a rate of 1.5°C min^−1^; the temperature was then raised to 86°C at a rate of 0.5°C min^−1^ and finally to 200°C at a rate of 30°C min^−1^; and the injection volume was 3 μl.
(PDF)
