# Supplementary material for: Efficient 2,3-Butanediol Production from Cassava Powder by a Crop-Biomass-Utilizer, Enterobacter cloacae subsp. dissolvens SDM
Source: PLoS One. 2012 Jul 5;7(7):e40442. doi: 10.1371/journal.pone.0040442 (PMC3390385; doi:10.1371/journal.pone.0040442)
Supplement: Figure S1 — Effects of temperature on cell growth and BD production. ΔOD620: the cell growth, which was determined by the difference value between the measured optical density in the fermentation process and the initial value at the beginning of the fermentation. RG: residual glucose. BD: 2,3-butanediol concentration. Data are the means ± SDs from three parallel experiments. Fermentations were carried out in 500-ml flasks with 100 ml of medium. (PDF) [file pone.0040442.s001.pdf]

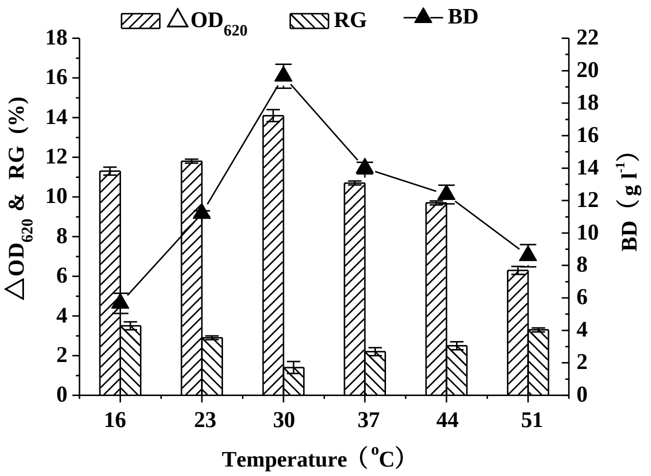

Figure S1. Effects of temperature on cell growth and BD production. ΔOD_{620}: the cell growth, which was determined by the difference value between the measured optical density in the fermentation process and the initial value at the beginning of the fermentation. RG: residual glucose. BD: 2,3-butanediol concentration. Data are the means ± SDs from three parallel experiments. Fermentations were carried out in 500-ml flasks with 100 ml of medium.
