# Supplementary material for: Efficient 2,3-Butanediol Production from Cassava Powder by a Crop-Biomass-Utilizer, Enterobacter cloacae subsp. dissolvens SDM
Source: PLoS One. 2012 Jul 5;7(7):e40442. doi: 10.1371/journal.pone.0040442 (PMC3390385; doi:10.1371/journal.pone.0040442)
Supplement: Figure S2 — Effects of the initial pH of the medium on cell growth and BD production. Data are the means ± SDs from three parallel experiments. The medium consisted of cassava powder hydrolysate (glucose concentration in the medium is 50 g l−1), peptone 5 g l−1, yeast extract 5 g l−1, sodium acetate 4 g l−1, KCl 0.5 g l−1, MgSO4·7H2O 0.15 g l−1, FeSO4·7H2O 0.05 g l−1, MnSO4·7H2O 0.03 g l−1. Fermentations were carried out in 500-ml flasks with 100 ml of medium at 30°C. (PDF) [file pone.0040442.s002.pdf]

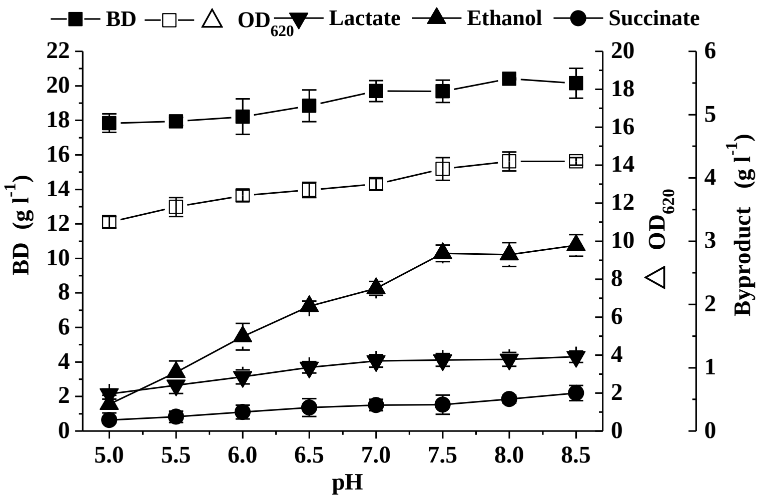

Figure S2. Effects of the initial pH of the medium on cell growth and BD production. Data are the means ± SDs from three parallel experiments. The medium consisted of cassava powder hydrolysate (glucose concentration in the medium is 50 g l^{-1}), peptone 5 g l^{-1}, yeast extract 5 g l^{-1}, sodium acetate 4 g l^{-1}, KCl 0.5 g l^{-1}, $MgSO_4\cdot 7H_2O$ 0.15 g l^{-1}, $FeSO_4\cdot 7H_2O$ 0.05 g l^{-1}, $MnSO_4\cdot 7H_2O$ 0.03 g l^{-1}. Fermentations were carried out in 500-ml flasks with 100 ml of medium at 30°C.
