# Supplementary material for: Efficient 2,3-Butanediol Production from Cassava Powder by a Crop-Biomass-Utilizer, Enterobacter cloacae subsp. dissolvens SDM
Source: PLoS One. 2012 Jul 5;7(7):e40442. doi: 10.1371/journal.pone.0040442 (PMC3390385; doi:10.1371/journal.pone.0040442)
Supplement: Figure S3 — GC analysis of fermented products by E. cloacae subsp. dissolvens SDM. IA: isoamyl alcohol was used as the internal standard. The ratio of the three stereoisomers of BD was analyzed by GC (Agilent GC6820) using a fused silica capillary column (Supelco Beta DEXTM 120, inside diameter, 0.25 mm; length, 30 m). The operating conditions were as follows: nitrogen was used as the carrier gas; the injector temperature and detector temperature were both 280°C; the column oven was maintained at 40°C for 3 min and then programmed to increase to 80°C at a rate of 1.5°C min−1; the temperature was then raised to 86°C at a rate of 0.5°C min−1 and finally to 200°C at a rate of 30°C min−1; and the injection volume was 3 μl. (PDF) [file pone.0040442.s003.pdf]

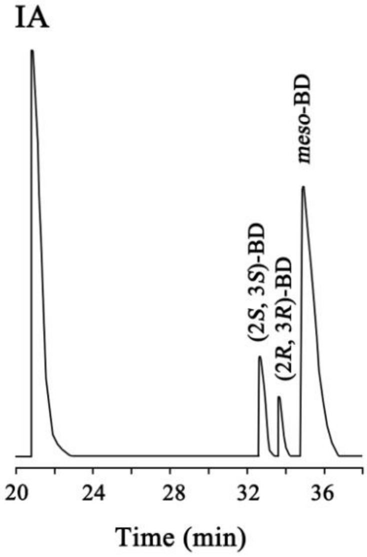

Figure S3. GC analysis of fermented products by *E. cloacae* subsp. *dissolvens* SDM. IA: isoamyl alcohol was used as the internal standard. The ratio of the three stereoisomers of BD was analyzed by GC (Agilent GC6820) using a fused silica capillary column (Supelco Beta DEX^{TM} 120, inside diameter, 0.25 mm; length, 30 m). The operating conditions were as follows: nitrogen was used as the carrier gas; the injector temperature and detector temperature were both 280°C; the column oven was maintained at 40°C for 3 min and then programmed to increase to 80°C at a rate of 1.5°C min^{-1}; the temperature was then raised to 86°C at a rate of 0.5°C min^{-1} and finally to 200°C at a rate of 30°C min^{-1}; and the injection volume was 3 μl.
